# Supplementary material for: Short-Term Benefits from Manual Therapy as an Adjunct Treatment for Persistent Postural-Perceptual Dizziness Symptoms: A Preliminary Prospective Case Series
Source: J Funct Morphol Kinesiol. 2024 May 3;9(2):82. doi: 10.3390/jfmk9020082 (PMC11130853; doi:10.3390/jfmk9020082)
Supplement: Supplementary file 1 [file jfmk-09-00082-s001.zip › jfmk-2953605-supplementary.pdf]

Table S1: Raw Scores: Before (Pre) and After (Post) Intervention Outcome Measures

			**DHI**		**TUG**		**Tandem (Pre)**		**Tandem (Post)**		**SLS (Pre)**		**SLS (Post)**	
**Patient**	**Age**	**# Visits**	**Pre**	**Post**	**Pre**	**Post**	**Left**	**Right**	**Left**	**Right**	**Left**	**Right**	**Left**	**Right**
1	72	4	58	20	12	10	15	12	15	12	6	8	11	13
2	83	8	44	16	13	10	5	3	12	12	0	0	4	7
3	76	7	44	12	16	11	6	7	15	13	7	5	10	12
4	86	3	36	12	13	13	2	4	5	7	0	0	4	4
5	71	5	60	8	10	9	10	10	10	14	8	6	12	11
6	79	3	42	10	16	11	3	0	6	3	0	0	0	0
7	17	5	44	0	9	3	25	30	30	30	25	27	30	30
8	49	7	88	12	12	4	12	8	30	30	3	3	30	30
9	78	3	60	12	10	8	0	5	22	14	3	5	16	17
10	56	3	56	12	8	6	7	0	17	14	16	17	19	16
11	79	4	56	8	9	9	3	3	15	15	5	10	10	12
12	73	2	58	0	12	8	10	14	25	22	10	8	18	16

Note: Total Dizziness Handicap Index (DHI) scores; Timed Up and Go (TUG) in seconds; Age (years); # = Number; Post = post-test; SLB = Single Leg-Stance in seconds; Tandem in seconds
